# Investigating potential drivers of increased central-line–associated bloodstream infections during the SARS-CoV-2 omicron-variant surge

**DOI:** 10.1017/ash.2023.284

**Published:** 2023-09-29

**Authors:** HeeEun Kang, Kathleen O. Stewart, Asif Khan, Stephanie C. Casale, Caitlin Adams Barker, Justin Kim

## Abstract

**Background:** Central-line–associated bloodstream infection (CLABSI) rates increased nationally during COVID-19, the drivers of which are still being characterized in the literature. CLABSI rates doubled during the SARS-CoV-2 omicron-variant surge at our rural academic medical center. We sought to identify potential drivers of CLABSIs by comparing period- and patient-specific characteristics of this COVID-19 surge to a historical control period. **Methods:** We defined the study period as the time of highest COVID-19 burden at our hospital (July 2021–June 2022) and the control period as the previous 2 years (July 2019–June 2021). We compared NHSN CLABSI standardized infection ratios (SIRs), central-line standardized utilization ratios (SURs), completion of practice evaluation tools (PETs) for monitoring of central-line bundle compliance, and proportions of traveling nurses. We performed chart reviews to determine patient-specific characteristics of NHSN CLABSIs during these periods, including demographics, comorbidities, central-line characteristics and care, and microbiology. **Results:** The CLABSI SIR was significantly higher during the study period than the control period (0.89 vs 0.52; *P* = .03); the SUR was significantly higher during the study period (1.08 vs 1.02; *P* < .01); the PET completion per 100 central-line days was significantly lower during the study period (23.0 vs 31.5; *P* < .01); and the proportion of traveling nurses was significantly higher during the study period (0.20 vs 0.08; *P* < .01) (Fig. 1). Patients with NHSN CLABSIs during the study period were more likely to have a history of COVID-19 (27% vs 3%; *P* = .01) and were more likely to receive a higher level of care (60% vs 27%; *P* = .02). During the study period, more patients had multilumen catheters (87% vs 61%; *P* = .04). The type of catheter, catheter care (ie, dressing changes and chlorhexidine bathing), catheter duration before CLABSI, and associated microbiology were similar between the study and control periods (Table 1). **Conclusions:** During the SARS-CoV-2 omicron-variant surge, the increase in CLABSIs at our hospital was significantly associated with increased central-line utilization, decreased PET completion, and increased proportion of traveling nurses. Critical illness and multilumen catheters were significant patient-specific factors that differed between CLABSIs from the study and control periods. We did not observe differences in catheter type, duration, or catheter care. Our study highlights key modifiable risk factors for CLABSI reduction. These findings may be surrogates for other difficult-to-measure challenges related to the culture of safety during a global pandemic, such as staff education related to infection prevention and daily review of central-line necessity.

**Figure:**
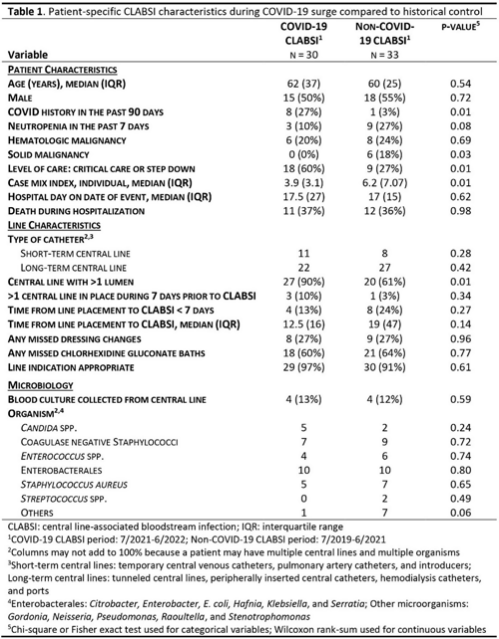

**Figure:**
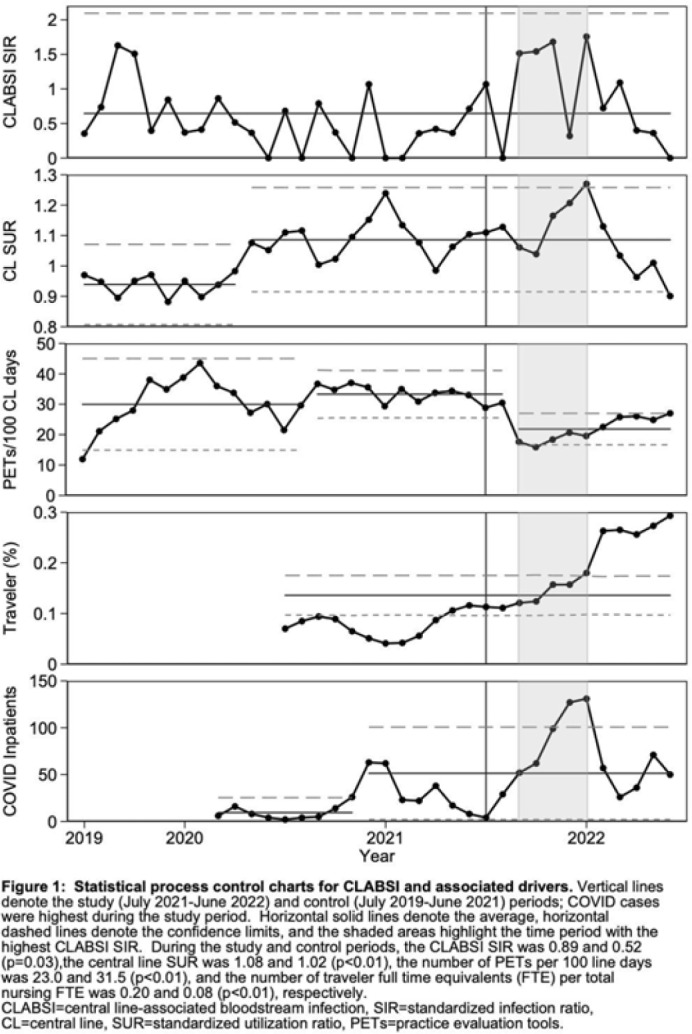

**Disclosures:** None

